# Modified Completion Test (MCT) in Psychological Diagnostics of Patients with Paranoid Schizophrenia — Stage of Filling the Gaps

**DOI:** 10.1192/j.eurpsy.2022.307

**Published:** 2022-09-01

**Authors:** N. Burlakova

**Affiliations:** Lomonosov Moscow State University, Faculty of Psychology, Department Of Neuro- And Pathopsychology, Moscow, Russian Federation

**Keywords:** thought disorder, cognitive assessment, schizophrénia, cognitive functions

## Abstract

**Introduction:**

The study demonstrates potential of the modified completion test (MCT) (text by H. Ebbinghaus) for diagnostics of patients with schizophrenia. MCT includes four stages: 1) filling the gaps in the story; 2) reading and retelling; 3) making up a continuation and a title; 4) retelling the story and its continuation after half an hour (Burlakova,2020).

**Objectives:**

The objective was to research diagnostical potential of the first stage of MCT for patients suffering from paranoid schizophrenia with hallucinatory syndrome.

**Methods:**

The study included 42 patients (28 female, 14 male) with schizophrenia (disease onset at least 5–7 years ago), aged from 19 to 51 (average age 35±8), receiving treatment. Control group consisted of 44 people (average age 37±6), never sought psychiatric help, never diagnosed with any mental disorders. Groups were organized to be equal in gender proportions, age, and educational level.

**Results:**

The psychiatric patients in comparison to the control group: 1) accomplished the task slower; 2) although instructed to fill the gaps in succession, often violated the instruction and demonstrated orientation on specific fragments rather than on the whole; 3) had lower efficiency: ˜5% of the clinical group did the task without mistakes; 4) chose strategies of interacting with the text not detected in the control group: a) did not fill several gaps, b) added words outside the gaps, and c) crossed out fragments of the text; 5) filled the gaps with words inadequate emotionally, semantically and/or logically.

**Conclusions:**

Comparative analysis demonstrated that already on the first stage, the method proves informative in pathopsychological assessment.

**Disclosure:**

No significant relationships.

